# Don’t Overshoot

**Published:** 1874-12

**Authors:** 


Don't Overshoot.—We are told that a Pittsburgh preacher uttered a sermon a few Sabbaths ago which sounded well, but which nobody understood. Accordingly, he has been requested to repeat it, and "say it slow." In one of his sentences he remarks: "The marvelous multitudinousness of the minutiæ of the corroborating circumstances are the insurmountable difficulties which unmistakably prevent the sceptic from discovering truth." But suppose that the circumambient nebulousness of the negations which would nullify the nonentities of sceptical cogitations was enlightened by the irridescent irradiations of clarified and glorified intuitions—what then? The trouble with too many speakers and writers is that they think more of sustaining their own reputation for scholarship than they do of the people. Simple language is the best, and wins its way in the long run.

